# Fournier´s Gangrene Under Sodium-Glucose Cotransporter 2 Inhibitor Therapy as a Life-Threatening Adverse Event: A Case Report and Review of the Literature

**DOI:** 10.7759/cureus.5778

**Published:** 2019-09-26

**Authors:** Severin Rodler, Thomas Weig, Christa Finkenzeller, Christian Stief, Michael Staehler

**Affiliations:** 1 Department of Urology, University Hospital, Ludwig Maximilian University of Munich, Munich, DEU; 2 Department of Anesthesiology, University Hospital, Ludwig Maximilian University of Munich, Munich, DEU; 3 Hospital Pharmacy, University Hospital, Ludwig Maximilian University of Munich, Munich, DEU

**Keywords:** fournier's gangrene, sglt2 inhibitor, dapagliflozin, type 2 diabetes mellitus, urinary tract infections

## Abstract

Sodium-glucose cotransporter 2 (SGLT2) inhibitors are a relatively new class of oral antidiabetic drugs. The US FDA has recently published a new warning about the safety of SGLT2 inhibitor administration in type 2 diabetes mellitus patients. There is an emerging evidence of an increased risk for developing Fournier´s gangrene (FG; a life-threatening complication) while under SGLT2 therapy. However, there are only three case reports and a total of 55 patients reported by the FDA to date. Therefore, there is a lack of evidence-based treatment algorithms for clinicians.

We present the case of a 39-year-old male patient with diabetes on oral dapagliflozin, metformin, and sitagliptin therapy who was admitted with FG to our hospital. Following emergency scrotal surgery, he had to be transferred to the intensive care unit due to respiratory and circulatory insufficiency. After a prolonged 27-day hospital stay with delirium, blood glucose imbalance, and five further surgical interventions, the patient was stabilized and discharged. Dapagliflozin was discontinued permanently.

This case demonstrates the risks of SGLT2 inhibitor therapy and the importance of early discontinuation after the occurrence of severe adverse events such as FG. According to the evidence in the literature, deranged glucose levels before admission are a common risk factor. However, further studies are required to identify patients at risks for FG and to investigate a direct connection with SGLT2 inhibitors.

## Introduction

Fournier´s gangrene (FG) is a rare, life-threatening necrotizing infection of the perineum or external genitalia [1]. As mortality rates are high, FG requires immediate surgical and intensive care treatment. Early debridement of all necrotic tissue, broad-spectrum antibacterial therapy, and hemodynamic support are important to generate favorable outcomes [2]. FG is mainly a clinical diagnosis. Patients with necrotizing fasciitis most commonly present with swelling, pain, and erythema [3]. In addition, laboratory results and imaging can be used to determine the severity of the infection and the extent of infected tissue [4]. Although FG is considered to be idiopathic by definition, risk factors to develop FG have been identified, including diabetes mellitus, alcohol abuse, poor hygiene, malignancies, and HIV infections. Most risk factors share features of immunosuppression and impairment in the microcirculation [5].

Sodium-glucose cotransporter 2 (SGLT2) inhibitors are a relatively new class of orally administered antidiabetic drugs first approved by the European Medicines Agency in 2012 and FDA in 2013 [6]. They inhibit the reabsorption of glucose from the urine by binding to SGLT2 in the proximal tubules of the kidney. This mechanism is independent of insulin, and therefore makes it an interesting drug in combination therapies for type 2 diabetes mellitus [7]. As of today, the FDA has approved four SGLT2 inhibitors: canagliflozin, dapagliflozin, empagliflozin, and ertugliflozin [8]. Patient outcomes are promising, as studies reveal significantly lower rates of cardiovascular events [9]. Side effects and complications can be considered severe. There are reports of ketoacidosis, acute kidney injury, and increased amputation rates [9-11]. Interestingly, urinary tract infections have been described to be significantly elevated under SGLT2 therapy as well, since SGLT2 inhibitors promote high glucose levels in the urine [12,13].

The FDA has recently published a warning about the occurrence of FG for patients under SGLT2 inhibitor therapy [14]. A patient with FG was treated in the intensive care unit of our hospital.

## Case presentation

A 39-year-old male patient was admitted to the emergency department of our hospital. At admission, the patient complained about having a fever for one week, as well as increased swelling and pain in the groin and testicles. As there was reported pus discharge the day before admission, the patient was started on azithromycin by a local physician. The patient´s history revealed type 2 diabetes mellitus, adipositas per magna, hypertension, nicotine abuse, an appendectomy, knee surgery, and allergies against dogs, cats, and grass. A history of oral antidiabetic medication for four years (metformin, sitagliptin, and dapagliflozin), antihypertensive therapy for four years (valsartan and amlodipine), and thyroid hormone replacement therapy for two years was stated by the patient. At presentation, the patient was clinically distressed but stable (blood pressure resting rate 146/74 mmHg, SpO_2_ 98%, breathing frequency 19 breaths per minute) with an increased heart rate (134 beats per minute) and fever (38.9°C). 

**Figure 1:**
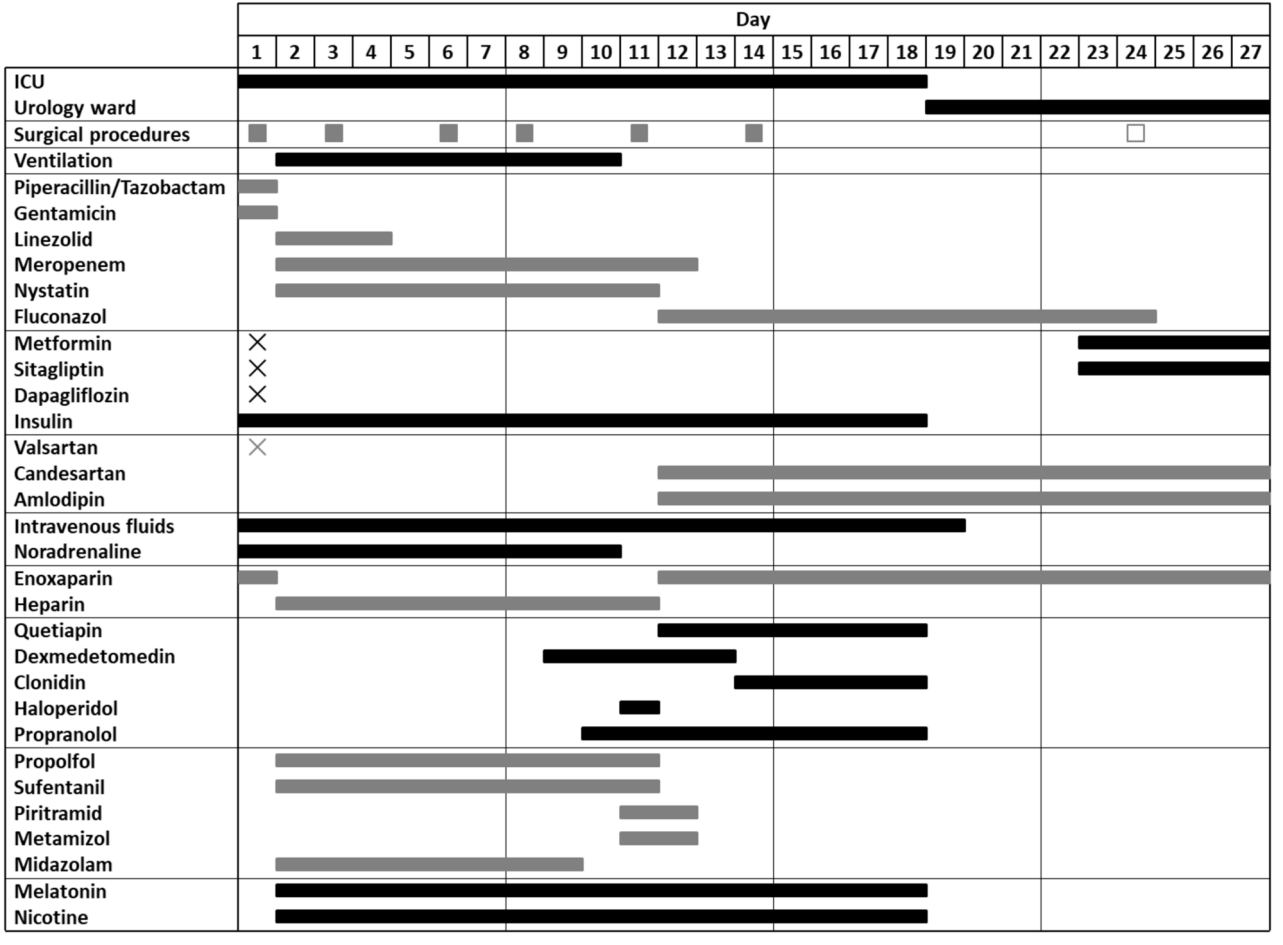
Timeline of surgical procedures, drugs, and supportive care. Treatment principles of early surgical intervention, broad antibacterial therapy, and hemodynamic support were followed. In addition to the stated drugs, hydrocortisone, pantoprazole, naloxone, furosemide, torsemide, urapidil, macrogol, sodium picosulfate, acetylcysteine, potassium chloride, cholecalciferol, thiamine, vitamin supplements, and enteral nutrition (Fresubin®) were administered. X indicates a discontinuation of the respective drug. ICU: intensive care unit.

During the physical examination, a swelling in the right groin and an intense smell was observed. The ultrasound examination showed normally perfused testicles and several abscesses in the groin. The results of the laboratory tests showed elevated inflammatory markers (IL-6: 383 pg/ml; C-reactive protein: 45.7 mg/dl; white blood cell count: 38.6 g/l), blood glucose of 302 mg/dl, and glucose as well as ketone bodies in the urine but no signs of a urinary tract infection. Blood and urine cultures were obtained. In the emergency room, a Foley catheter was inserted, and broad-spectrum antibiotic therapy was added (160 mg gentamicin and 4 g piperacillin with 0.5 g tazobactam) given intravenously. As supportive measures, intravenous fluid therapy and enoxaparin 0.8 mg twice per day were started. All oral antidiabetic drugs were stopped at admission, and the patient was started on a basal-bolus insulin treatment in the emergency room (Figure 1).

**Figure 2:**
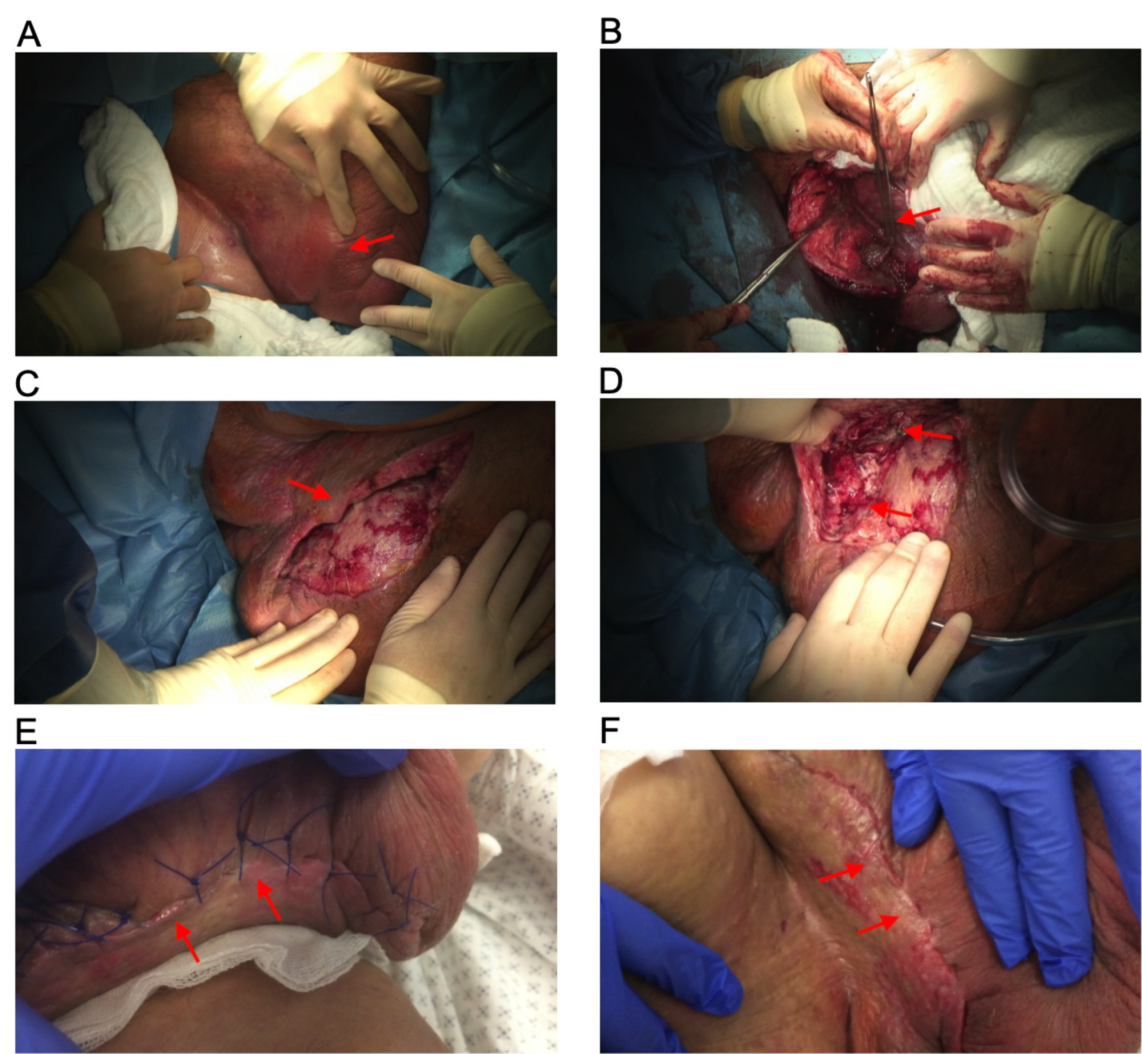
Images of groin and scrotum. A: Day one. The images acquired before the initial surgery showed massive swelling in the groin and scrotum. B: On day one, necrotic tissue was removed intraoperatively. C, D: On day eight, further debridement of necrotic tissue was performed. The extend of debridement is depicted in the images shown. E: On day 24, the wound has already healed, and stitches were removed. F: After stitches had been removed, an excellent cosmetic result is observed on day 27.

The patient was immediately transferred to the operating room, where the abscess and necrosis of the scrotum, the groin, and perineum were resected, and an easy-flow drainage was put in place (Figure 2). Due to the perioperative onset of septic shock with a high need for catecholamine (1 mg/h noradrenaline) and respiratory insufficiency, the patient was admitted to the intensive care unit (ICU). In the ICU, a calculated enhanced antibiotic treatment regime of meropenem and linezolid was added. On day three, a second-look procedure with debridement was performed. Meanwhile, the results from the culture of smears from the groin and scrotum were positive for *Peptostreptococcus anaerobius*. The blood cultures remained negative for aerobic and anaerobic species. On day six, a vacuum-assisted closure system was installed and changed on day eight (accompanied by further debridement) and day 11. After receiving cultures from the follow-up smears from the groin and scrotum positive for *Candida albicans*, intravenous fluconazole was started. On day 14, the wound was closed. The ICU stay was further complicated by delirium and derailed diabetes mellitus.

On day 18, the patient was transferred from the ICU to a normal urology ward. Here, he was successfully mobilized, and the antidiabetic treatment of metformin and sitagliptin was started again. Dapagliflozin was not restarted. Under this treatment, the patient successfully recovered and was discharged on day 27.

## Discussion

To date, only three case reports of FG occurring in patients under SGLT2 therapy have been published (Table 1). In total, there are 55 patients reported to the FDA who have suffered FG under this antidiabetic medication [14].

**Table 1:** Patient characteristics of available case reports HbA1c: glycated hemoglobin; ICU: intensive care unit; n.a.: not applicable. *Abstract: Chi WC, Lim-Tio S. Fournier’s Syndrome: A Life-Threatening Complication of SGLT2 Inhibition in Poorly Controlled Diabetes Mellitus. 2016 Joint Annual Scientific Meeting of the Australian Diabetes Educators Association (ADEA) and the Australian Diabetes Society (ADS); August 25, 2016.

Age (years)	Drug name	Initiation	HbA1c	BMI	Comorbidities	Hospital	ICU	Reference
67	Dapagliflozin	3 weeks	10.8%	n.a.	Obesity	51	n.a.	*
41	Empagliflozin	7 months	11.2%	38	Obesity, smoking	15	n.a.	[15]
60	Dapagliflozin	4 months	n.a.	n.a.	n.a	12	6	[16]
39	Dapagliflozin	4 years	10.0%	49	Obesity, smoking, hypertension	27	18	This paper

Chi et al. reported a 67-year-old male patient with poorly controlled type 2 diabetes mellitus (glycated hemoglobin [HbA1c], 10.8%) who was on dapagliflozin therapy and was required multiple surgeries to treat his FG. Dapagliflozin was ceased, and basal insulin therapy was started after admission. In total, he stayed hospitalized for 51 days (Abstract: Chi WC, Lim-Tio S. Fournier’s Syndrome: A Life-Threatening Complication of SGLT2 Inhibition in Poorly Controlled Diabetes Mellitus. 2016 Joint Annual Scientific Meeting of the Australian Diabetes Educators Association (ADEA) and the Australian Diabetes Society (ADS); August 25, 2016).

Kumar et al. reported a 41-year-old male patient with type 2 diabetes mellitus who was on an empagliflozin and metformin-based regime with an elevated HbA1c of 11.2 %. The patient required multiple surgeries and was admitted for 15 days to the hospital [15].

Omer et al. reported a 60-year-old male patient with type 2 diabetes mellitus who was on dapagliflozin for four months before he developed FG. After six days in the ICU and a total of 12 days in the hospital, he was discharged [16].

SGLT2 inhibitors are prescribed to 1.7 million American patients [14]. In contrast, the number of reported cases with FG is still small. Also, the reported cases show no clear correlation between therapy initiation and the onset of FG. Therefore, the comorbidities of the reported patients have to be considered.

All patients shared a poorly adjusted diabetes mellitus at the time of admission save for the patient of Omer et al. whose HbA1c value was not reported. The three other patients presented with HbA1c values of at least 10%. Using published algorithms, this means that they have had an average glucose level of 13.4 mmol/l during the weeks before admission [17].

Obesity was a common feature of the three patients but not reported for the patient published by Omer et al. Our patient and the patient of Kumar et al. reported smoking which affects the microcirculation, a previously mentioned risk factor for FG [5]. 

As FG is mainly a clinical diagnosis, special attention in urological examinations of patients with a history of SGLT2 use and poorly adjusted glucose levels should be made for signs of FG. Relevant comorbidities such as obesity and a history of smoking as well as unclear or previous genital infections should alert the clinicians to focus on checking for this life-threatening disease.

The initial treatment algorithm in all cases was based on radical debridement, broad-spectrum antibiotics, and supportive measures such as hemodynamic management, which is commonly agreed on as the treatment of FG [2]. Oral antidiabetic therapy was stopped directly after admission by Kumar et al., Chi et al., and in our case report.

There is no evidence so far regarding the reapplication of SGLT2 inhibitors after FG. It is unclear whether patients who have suffered once from FG should not be started on SGLT2 inhibitors again after complete remission. We decided not to reapply dapagliflozin in our case.

However, the question remains whether SGLT2 inhibitors are a direct cause of FG or are just correlated with the comorbidities. According to the FDA, there have been only 19 cases of FG in 35 years, which were associated with other oral antidiabetic drugs other than SGLT2 inhibitors. However, within just six years of market authorization for SGLT2 inhibitors, there have already been reported 55 cases of FG associated with this therapy [14]. This accumulation of cases in a short time is suggestive of a correlation. The underlying mechanism is unclear. Potentially, deranged glucose levels in the blood and additional SGLT2 therapy may lead to extremely elevated glucose levels in the urine. This provides a favorable milieu for urinary infections. Together with local immunodeficiency (caused by diabetes mellitus and other comorbidities) and impaired microvascular supply, this may cause FG in a certain subpopulation of patients.

The described cases and findings from the FDA contrast with the existing randomized controlled studies and safety profiles for SGLT2 inhibitors. However, it is a known fact that extremely rare adverse effects might be missed during pivotal trials and are revealed only in a bigger patient population after approval. Acidosis and the risk of bone fracture under SGLT2 therapy is a comparably rare event as well and was not reported until after market authorization [18]. Furthermore, phase IV studies are required to investigate these extremely rare adverse events and to better understand the safety profiles and identify patients at risk. This case report adds important insights towards the understanding of these adverse events as it links the risk of FG to deranged glucose levels in patients under SGLT-2 inhibitor therapy.

Besides the discussed SGLT2 inhibitors, dual SGLT1/SGLT2 inhibitors are emerging. SGLT1 is expressed predominantly in the intestines and the late proximal tubules of the kidney. Due to its position downstream of SGLT2, the effects of SGLT1 inhibition in the kidney are mainly observed when SGLT2 glucose reabsorption is insufficient or blocked [19]. Considering the supposed connection between deranged glucose levels in the blood and urine with FG development, side effects of this new drug class have to be monitored cautiously in early trials. 

## Conclusions

We presented a patient who developed FG while under SGLT2 inhibitor therapy. After a literature review and considering the recently published warning by the FDA, a link between SGLT2 inhibitors and FG can be suspected. As three patients in the literature presented with highly elevated HbA1c levels at admission, a deranged glucose level leading to high glucose levels in the urine under SGLT2 inhibitor therapy has been identified as a risk factor. Further studies are necessary to determine risk factors, generate evidence for a causal connection, and to improve treatment algorithms for patients with FG.

## References

[1] Hagedorn JC, Wessells H (2017). A contemporary update on Fournier’s gangrene. Nat Rev Urol.

[2] Furr J, Watts T, Street R, Cross B, Slobodov G, Patel S (2017). Contemporary trends in the inpatient management of Fournier’s gangrene: predictors of length of stay and mortality based on population-based sample. Urology.

[3] Goh T, Goh LG, Ang CH, Wong CH (2014). Early diagnosis of necrotizing fasciitis. Br J Surg.

[4] Voelzke BB, Hagedorn JC (2018). Presentation and diagnosis of Fournier gangrene. Urology.

[5] Chennamsetty A, Khourdaji I, Burks F, Killinger KA (2015). Contemporary diagnosis and management of Fournier’s gangrene. Ther Adv Urol.

[6] Haas B, Eckstein N, Pfeifer V, Mayer P, Hass MDS (2014). Efficacy, safety and regulatory status of SGLT2 inhibitors: focus on canagliflozin. Nutr Diabetes.

[7] Ghosh RK, Ghosh SM, Chawla S, Jasdanwala SA (2012). SGLT2 inhibitors: a new emerging therapeutic class in the treatment of type 2 diabetes mellitus. J Clin Pharmacol.

[8] Powell J, Garland SG (2019). Ertugliflozin: a new option in the sglt-2 inhibitor market for the treatment of type 2 diabetes mellitus. Ann Pharmacother.

[9] Neal B, Perkovic V, Mahaffey KW (2017). Canagliflozin and cardiovascular and renal events in type 2 diabetes. N Engl J Med.

[10] Fralick M, Schneeweiss S, Patorno E (2017). Risk of diabetic ketoacidosis after initiation of an SGLT2 inhibitor. N Engl J Med.

[11] Hahn K, Ejaz AA, Kanbay M, Lanaspa MA, Johnson RJ (2016). Acute kidney injury from SGLT2 inhibitors: potential mechanisms. Nat Rev Nephrol.

[12] Liu J, Li L, Li S (2017). Effects of SGLT2 inhibitors on UTIs and genital infections in type 2 diabetes mellitus: a systematic review and meta-analysis. Sci Rep.

[13] Zinman B, Wanner C, Lachin JM (2015). Empagliflozin, cardiovascular outcomes, and mortality in type 2 diabetes. N Engl J Med.

[14] Bersoff-Matcha SJ, Chamberlain C, Cao C, Kortepeter C, Chong WH (2019). Fournier gangrene associated with sodium-glucose cotransporter-2 inhibitors: a review of spontaneous postmarketing cases. Ann Intern Med.

[15] Kumar S, Costello AJ, Colman PG (2017). Fournier’s gangrene in a man on empagliflozin for treatment of type 2 diabetes. Diabet Med.

[16] Omer TAM, Dharan SS, Adler A (2018). Sodium‐glucose cotransporter 2 (SGLT‐2) inhibitor dapagliflozin and Fournier’s gangrene: a life‐threatening severe adverse outcome. Case report. Diabet Med.

[17] Nathan DM, Kuenen J, Borg R, Zheng H, Schoenfeld D, Heine RJ (2008). Translating the A1C assay into estimated average glucose values. Diabetes Care.

[18] Wu JHY, Foote C, Blomster J (2016). Effects of sodium-glucose cotransporter-2 inhibitors on cardiovascular events, death, and major safety outcomes in adults with type 2 diabetes: a systematic review and meta-analysis. Lancet Diabetes Endocrinol.

[19] Song P, Onishi A, Koepsell H, Vallon V (2016). Sodium glucose cotransporter SGLT1 as a therapeutic target in diabetes mellitus. Expert Opin Ther Targets.

